# Supplementary material for: Influence of Prevalence of Psychoactive Substance Use in Mexican Municipalities on Early Childhood Development
Source: Int J Environ Res Public Health. 2021 Sep 24;18(19):10027. doi: 10.3390/ijerph181910027 (PMC8507637; doi:10.3390/ijerph181910027)
Supplement: Supplementary file 1 [file ijerph-18-10027-s001.zip › ijerph-1250224-supplementary.pdf]

Supplement File

Title: Influence of Prevalence of Psychoactive Substance Use in Mexican Municipalities on Early Childhood Development.

Authors:

Francisco-Javier Prado-Galbarro, Copytzy Cruz-Cruz, Jorge-Ameth Villatoro-Velázquez and Juan-Manuel Martínez-Núñez*

* Correspondence: Dr. Juan-Manuel Martínez-Núñez, Orphan Drug Laboratory, Biologic System Department, Universidad Autónoma Metropolitana Campus Xochimilco, Mexico City 04960, Mexico; jmartinezn@correo.xoc.uam.mx

Table of Contents

Supplement Table S1. Individual Characteristics for Children with Inadequate or Adequate Early Childhood Development by Domains.

Supplement Table S2. Prevalence of Psychoactive Substance Use and Environmental Characteristics of the Municipalities where Children with Inadequate or Adequate Early Childhood Development live by Domains.

Supplement Table S1. Individual Characteristics for Children with Inadequate or Adequate Early Childhood Development by Domains.

	Socio-emotional development			Literacy-numeracy			Learning			Physical development		
	**Inadequate**	**Adequate**	**P-value**	**Inadequate**	**Adequate**	**P-value**	**Inadequate**	**Adequate**	**P-value**	**Inadequate**	**Adequate**	**P-value**
**Functional difficulties, %**												
No	19.62 (15.86 - 24.01)	80.38 (75.99 - 84.14)	0.067	73.89 (68.04 - 79)	26.11 (21.00 - 31.96)	0.022	1.96 (1.30 - 2.93)	98.04 (97.07 - 98.7)	0.014	1.49 (0.97 - 2.27)	98.51 (97.73 - 99.03)	0.148
Yes	35.74 (18.86 - 57.09)	64.26 (42.91 - 81.14)		90.52 (76.84 - 96.49)	9.48 (3.51 - 23.16)		7.34 (2.54 - 19.41)	92.66 (80.59 - 97.46)		4.26 (1.04 - 15.86)	95.74 (84.14 - 98.96)	
**Mother's education, %**												
Primary or less	22.37 (16.07 - 30.25)	77.63 (69.75 - 83.93)	0.03	81.08 (74.33 - 86.38)	18.92 (13.62 - 25.67)	0.111	4.16 (2.23 - 7.42)	95.84 (92.58 - 97.70)	0.057	1.54 (0.56 - 4.16)	98.46 (95.84 - 99.44)	0.706
Middle school	24.43 (19.22 - 30.52)	75.57 (69.48 - 80.78)		72.17 (64.45 - 78.77)	27.83 (21.23 - 35.55)		2.10 (1.34 - 3.29)	97.90 (96.71 - 98.66)		1.90 (1.18 - 3.05)	98.10 (96.95 - 98.82)	
High school	19.12 (14.91 - 24.2)	80.88 (75.80 - 85.09)		65.91 (58.07 - 72.97)	34.09 (27.03 - 41.93)		1.48 (0.74 - 2.96)	98.52 (97.04 - 99.26)		1.28 (0.58 - 2.79)	98.72 (97.21 - 99.42)	
University	9.49 (4.02 - 20.82)	90.51 (79.18 - 95.98)		82.50 (64.59 - 92.42)	17.50 (7.58 - 35.41)		1.01 (0.25 - 3.92)	98.99 (96.08 - 99.75)		1.01 (0.25 - 3.92)	98.99 (96.08 - 99.75)	
**Wealth quintiles for households, %**												
Very poor	27.35 (19.08 - 37.54)	72.65 (62.46 - 80.92)	0.114	87.43 (80.87 - 91.96)	12.57 (8.04 - 19.13)	0.09	4.88 (2.27 - 10.20)	95.12 (89.80 - 97.73)	0.137	3.10 (1.38 - 6.8)	96.90 (93.20 - 98.62)	0.170
Poor	20.61 (15.96 - 26.18)	79.39 (73.82 - 84.04)		69.98 (61.20 - 77.51)	30.02 (22.49 - 38.80)		1.62 (0.90 - 2.90)	98.38 (97.10 - 99.10)		0.67 (0.27 - 1.67)	99.33 (98.33 - 99.73)	
Middle	21.49 (13.13 - 33.13)	78.51 (66.87 - 86.87)		72.82 (63.48 - 80.50)	27.18 (19.50 - 36.52)		1.93 (0.85 - 4.34)	98.07 (95.66 - 99.15)		2.14 (1.22 - 3.71)	97.86 (96.29 - 98.78)	
Rich	22.64 (16.34 - 30.47)	77.36 (69.53 - 83.66)		66.39 (54.76 - 76.32)	33.61 (23.68 - 45.24)		1.62 (0.73 - 3.54)	98.38 (96.46 - 99.27)		1.39 (0.60 - 3.15)	98.61 (96.85 - 99.4)	
Very rich	10.18 (4.63 - 20.91)	89.82 (79.09 - 95.37)		81.50 (64.30 - 91.50)	18.50 (8.50 - 35.70)		1.54 (0.48 - 4.85)	98.46 (95.15 - 99.52)		1.16 (0.31 - 4.18)	98.84 (95.82 - 99.69)	
**Sex, %**												
Boys	25.08 (21.63 - 28.87)	74.92 (74.92 - 74.92)	0.02	74.21 (68.80 - 78.97)	25.79 (25.79 - 25.79)	0.97	3.61 (2.41 - 5.36)	96.39 (96.39 - 96.39)	0	2.27 (1.44 - 3.55)	97.73 (97.73 - 97.73)	0.028
Girls	15.89 (10.86 - 22.68)	84.11 (77.32 - 89.14)		74.02 (64.61 - 81.64)	25.98 (18.36 - 35.39)		0.84 (0.41 - 1.73)	99.16 (98.27 - 99.59)		0.96 (0.5 - 1.87)	99.04 (98.13 - 99.5)	
**Indigenous.,%**												
No	20.48 (15.76 - 26.18)	79.52 (73.82 - 84.24)	0.655	74.85 (67.99 - 80.66)	25.15 (19.34 - 32.01)	0.71	1.91 (1.24 - 2.94)	98.09 (97.06 - 98.76)	0.571	1.45 (0.90 - 2.33)	98.55 (97.67 - 99.1)	0.413
Yes	18.72 (13.74 - 24.97)	81.28 (75.03 - 86.26)		72.74 (62.56 - 80.99)	27.26 (19.01 - 37.44)		2.42 (1.18 - 4.89)	97.58 (95.11 - 98.82)		2.09 (0.99 - 4.37)	97.91 (95.63 - 99.01)	
**Area, %**												

Urban	19.12 (15.03 – 24.00)	80.88 (76.00 - 84.97)	0.146	73.33 (66.70 - 79.05)	26.67 (20.95 - 33.30)	0.189	2.01 (1.30 - 3.09)	97.99 (96.91 - 98.70)	0.919	1.50 (0.96 - 2.33)	98.50 (97.67 - 99.04)	0.863
Rural	24.58 (19.07 - 31.06)	75.42 (68.94 - 80.93)		79.27 (72.33 - 84.83)	20.73 (15.17 - 27.67)		2.12 (0.86 - 5.15)	97.88 (94.85 - 99.14)		1.66 (0.57 - 4.72)	98.34 (95.28 - 99.43)	
**Domestic violence, %**												
No	15.82 (11.76 - 20.96)	84.18 (79.04 - 88.24)	0.147	68.83 (60.47 - 76.12)	31.17 (23.88 - 39.53)	0.129	1.61 (0.90 - 2.86)	98.39 (97.14 - 99.10)	0.374	1.26 (0.68 - 2.35)	98.74 (97.65 - 99.32)	0.490
Yes	21.83 (16.22 - 28.72)	78.17 (71.28 - 83.78)		76.75 (69.47 - 82.72)	23.25 (17.28 - 30.53)		2.24 (1.37 - 3.64)	97.76 (96.36 - 98.63)		1.65 (1.00 - 2.71)	98.35 (97.29 – 99.00)	
**Maternal age, mean (SD)**	28.09 (7.29)	30 (6.35)	0.007	29.86 (6.37)	28.92 (7.16)	0.286	29.58 (9.43)	29.62 (6.53)	0.978	28.88 (9.10)	29.63 (6.55)	0.621
**Children age (months), mean (SD)**	46.35 (8.06)	49.48 (6.64)	<0.001	48.07 (7)	51.14 (6.61)	0.001	41.23 (10.09)	49.02 (6.88)	<0.001	39.15 (7.88)	49.01 (6.91)	<0.001

* Significant value ($p < 0.05$).

Supplement Table S2. Prevalence of Psychoactive Substance Use and Environmental Characteristics of the Municipalities where Children with Inadequate or Adequate Early Childhood Development live by Domains.

Variable, mean (SD)	Socio-emotional development			Literacy-numeacy			Learning			Physical development		
	Inadequate	Adequate	P-value	Inadequate	Adequate	P-value	Inadequate	Adequate	P-value	Inadequate	Adequate	P-value
% Using illegal drugs	3.64 (2.47)	3.29 (2.1)	0.114	3.3 (2.15)	3.52 (2.27)	0.381	3.9 (2.69)	3.35 (2.17)	0.145	3.34 (1.87)	3.36 (2.18)	0.959
% Using non-prescription medical drugs	0.63 (0.95)	0.51 (1.08)	0.277	0.5 (0.97)	0.64 (1.29)	0.223	0.63 (1.22)	0.54 (1.06)	0.669	0.55 (1.05)	0.54 (1.06)	0.939
Marginalization index	-1.35 (0.56)	-1.4 (0.51)	0.247	-1.38 (0.51)	-1.4 (0.57)	0.667	-1.32 (0.6)	-1.39 (0.52)	0.511	-1.27 (0.61)	-1.39 (0.52)	0.261
Number of homicides	145.95 (199.28)	120.82 (153.01)	0.087	122.71 (156.54)	134.56 (178.4)	0.557	160.27 (231.37)	125.08 (160.95)	0.262	149.67 (252.39)	125.43 (161.08)	0.511
Population density by municipality	2210.35 (4233.03)	3205.35 (4686.72)	0.011	2690.05 (4178.49)	3918.07 (5730.08)	0.083	3139.52 (5439.93)	3005.37 (4631.69)	0.847	2929.98 (5260.98)	3009.29 (4639.99)	0.926
% Drug-dependent users	0.9 (1.19)	0.74 (0.96)	0.053	0.77 (1.01)	0.77 (1.01)	0.997	1.18 (1.86)	0.76 (0.99)	0.117	0.88 (1.3)	0.77 (1)	0.577
% Users without drug dependence	11.31 (4.35)	11.45 (4.47)	0.738	11.1 (3.83)	12.36 (5.87)	0.135	12.25 (5.4)	11.41 (4.45)	0.283	11.45 (4.93)	11.43 (4.46)	0.972
% Non users: With exposure to drugs	18.4 (6.08)	18.71 (5.97)	0.622	18.76 (6.2)	18.32 (5.44)	0.582	18.32 (5.79)	18.66 (6.01)	0.69	19.28 (6.26)	18.64 (6.01)	0.492
% Non users: No drug exposure	69.39 (7.16)	69.1 (6.68)	0.649	69.36 (6.75)	68.56 (6.9)	0.319	68.25 (7.95)	69.17 (6.76)	0.377	68.39 (7.66)	69.17 (6.78)	0.498

* Significant value ($p < 0.05$).
